# A novel inhibitory effect of oxazol-5-one compounds on ROCKII signaling in human coronary artery vascular smooth muscle cells

**DOI:** 10.1038/srep32118

**Published:** 2016-08-30

**Authors:** Abdulhameed Al-Ghabkari, Jing-Ti Deng, Paul C. McDonald, Shoukat Dedhar, Mana Alshehri, Michael P. Walsh, Justin A. MacDonald

**Affiliations:** 1 Department of Biochemistry & Molecular Biology, University of Calgary, 3280 Hospital Drive NW, Calgary, AB, T2N 4Z6, Canada; 2 Department of Integrative Oncology, BC Cancer Research Centre, 675 West 10th Ave, Vancouver, BC, V5Z 1L3, Canada

## Abstract

The selectivity of (4Z)-2-(4-chloro-3-nitrophenyl)-4-(pyridin-3-ylmethylidene)-1,3-oxazol-5-one (DI) for zipper-interacting protein kinase (ZIPK) was previously described by in silico computational modeling, screening a large panel of kinases, and determining the inhibition efficacy. Our assessment of DI revealed another target, the Rho-associated coiled-coil-containing protein kinase 2 (ROCKII). *In vitro* studies showed DI to be a competitive inhibitor of ROCKII (Ki, 132 nM with respect to ATP). This finding was supported by in silico molecular surface docking of DI with the ROCKII ATP-binding pocket. Time course analysis of myosin regulatory light chain (LC20) phosphorylation catalyzed by ROCKII *in vitro* revealed a significant decrease upon treatment with DI. ROCKII signaling was investigated *in situ* in human coronary artery vascular smooth muscle cells (CASMCs). ROCKII down-regulation using siRNA revealed several potential substrates involved in smooth muscle contraction (e.g., LC20, Par-4, MYPT1) and actin cytoskeletal dynamics (cofilin). The application of DI to CASMCs attenuated LC20, Par-4, LIMK, and cofilin phosphorylations. Notably, cofilin phosphorylation was not significantly decreased with a novel ZIPK selective inhibitor (HS-38). In addition, CASMCs treated with DI underwent cytoskeletal changes that were associated with diminution of cofilin phosphorylation. We conclude that DI is not selective for ZIPK and is a potent inhibitor of ROCKII.

Smooth muscle plays an important role in the regulation of vascular tone and many other biological functions. Of central importance to the development of vascular smooth muscle (VSM) tone is the variable nature of the relationship between cytosolic free Ca^2+^ concentration ([Ca^2+^]_*i*_) and force generation, with greater force achieved in the absence of a change in [Ca^2+^]_*i*_ through processes collectively referred to as ‘Ca^2+^ sensitization’^1^,^2^,^3^,^4^. To initiate contraction, an increase in [Ca^2+^]_*i*_ activates myosin light chain kinase (MLCK), a Ca^2+^/calmodulin-dependent enzyme. MLCK phosphorylates the regulatory light chains (LC20) of myosin II on Ser19, resulting in contraction of smooth muscle through increases in actin-activated myosin MgATPase activity and cross-bridge cycling^5^. Myosin light chain phosphatase (MLCP) is responsible for the dephosphorylation of LC20 resulting in relaxation of VSM^6^,^7^. Although a change in [Ca^2+^]_*i*_ is the primary determinant of VSM contraction, it is the balance between MLCK and MLCP activities that dictates the contractile activity of VSM. Indeed, MLCP functions independently of Ca^2+^-calmodulin and can be regulated by G protein-coupled receptors and downstream signaling modules. A variety of studies have demonstrated that MLCP activity (and hence Ca^2+^ sensitization) is regulated by a number of protein kinases that act to phosphorylate the myosin phosphatase-targeting subunit (MYPT1)^6^,^7^. MLCP activity can also be attenuated indirectly via the phosphorylation of the 17 kDa-protein inhibitor of MLCP, CPI-17^8^. An additional mechanism for Ca^2+^ sensitization is not dependent on MLCP inhibition but rather on the direct phosphorylation of LC20^5^,^9^: the Ca^2+^-independent diphosphorylation of LC20 at both Thr18 and Ser19 induces force comparable to that evoked by MLCK-catalyzed phosphorylation at Ser19, but force is sustained due to a reduction in the rate of dephosphorylation of diphosphorylated compared to monophosphorylated LC20^10^. It is likely, therefore, that a cooperative network of kinases contributes to regulate VSM tone during Ca^2+^ sensitization.

Several protein kinases are linked to the Ca^2+^ sensitization phenomenon in VSM, with a prominent contribution of Rho-associated coiled coil-containing kinase (ROCK) revealed in the literature^3^,^11^,^12^,^13^,^14^,^15^. ROCK is a well-characterized effector of the small GTPase RhoA and belongs to the AGC (protein kinases A, G and C) family of classical Ser/Thr protein kinases^15^. There are two members of the ROCK family, ROCKI (ROKβ or p160ROCK) and ROCKII (ROKα), and both members share significant conservation of sequence (92% identity in the kinase domain). In humans, both ROCKI and ROCKII are ubiquitously expressed across tissues. Both isoforms are expressed in smooth muscle with possible distinction in functions; however, ROCKII appears to provide critical regulation of VSM cells (VSMCs)^15^. In this regard, ROCKII is a key regulator of contractile actomyosin fibers and cytoskeletal dynamics. ROCKII is able to phosphorylate LC20^16^,^17^, MYPT1^12^,^14^,^18^ and CPI-17^19^. Prevailing evidence indicates, however, that ROCKII does not directly phosphorylate LC20 in smooth muscle tissues (discussed in ref. 20). Additionally, actin polymerization is regulated by RhoA/ROCKII activation of LIN-11, ISL1 and MEC-3 (LIM) kinase, leading to the inhibition of the actin-severing protein cofilin^15^,^21^,^22^.

The coordinated regulation of tone via protein kinases is a key functional property of VSM, and it is not surprising that VSMCs possess a variety of signal transduction mechanisms to regulate force development. Zipper-interacting protein kinase (ZIPK, also known as death-associated protein kinase 3, DAPK3) is a Ser/Thr protein kinase that has emerged as a key regulator of Ca^2+^ sensitization and VSMC contractility^23^,^24^,^25^,^26^,^27^,^28^,^29^ as well as vascular inflammatory responses^20^,^30^ and hypertrophic remodeling^31^. This kinase was implicated in the phosphorylation of LC20^24^,^25^,^29^,^32^,^33^, of MYPT1^23^,^26^,^27^, and of CPI-17^29^,^34^. Moreover, ZIPK is linked to a number of additional biological processes^35^,^36^,^37^, including apoptosis, cellular autophagy, chromatin structural changes, and reorganization of the actin cytoskeleton (which has been reported by the observation of membrane blebbing, cell rounding and detachment from the cell matrix by over-expression of ZIPK in non-muscle cells^32^,^33^).

The potential cross-talk between ZIPK and ROCK activities^38^ and the overlap in the physiological functions of the two kinases in VSM required development of a selective inhibitor to distinguish between these kinases^39^. Specific small molecule inhibitors of ZIPK were not available until recently; however, three independent groups have now reported novel molecular inhibitor scaffolds: oxo-β-carbolines^40^, benzylidene oxazolones^41^,^42^, and thiol-substituted pyrazolo[3,4-d]pyrimidinones^43^. The *in vitro* characterization of the 2-phenyl-4-(3-pyridinylmethylene)-5(4H)-oxazolone inhibitor (also known as DI) suggested specificity and potency of this compound toward ZIPK^41^,^42^. More recent applications of the DI compound in VSM revealed novel functions of ZIPK in (i) development of essential hypertension in mesenteric arteries of the spontaneously hypertensive rat^31^, and vascular inflammatory and hypertrophic remodeling responses^44^. Based on these data, we employed the DI compound in studies to define the functional targets of ZIPK in human coronary artery VSMCs, and confirmed that the DI compound possesses potent inhibitory action against ZIPK. However, our interrogations revealed unreported off-target activity toward the important VSM contractile kinase, ROCKII. As such, it is difficult to identify whether biological effects of DI are restricted to ZIPK, a secondary effect of ROCKII signal attenuation, or some combination of the two. Although there is potential value for the DI compound to study the biological actions of ZIPK, our results suggest the overlapping functions of ROCKII and ZIPK in VSMC contractile mechanisms preclude the use of DI in this context.

## Experimental Procedures

### Materials

The DAPK3/ZIPK inhibitors (4Z)-2-(4-chloro-3-nitrophenyl)-4-(pyridin-3-ylmethylidene)-1,3-oxazol-5-one (DI, PubChem CID: 6516567), (4Z)-2-ethylidene(phenyl)-4-(pyridin-3-ylmethylidene)-1,3-oxazol-5-one (DI-2, PubChem CID: 2360773), and (4Z)-2-(3-methylphenyl)-4-(pyridin-3-ylmethylidene)-1,3-oxazol-5-one (DI-3, PubChem CID: 5285453) were purchased from ChemDiv (San Diego, CA). The ROCK inhibitors, GSK269962A (PubChem CID: 16095342) and H1152 (PubChem CID: 448043), along with staurosporine, were acquired from Alexis Biochemicals (San Diego, CA). HS-38 (PubChem CID: 42938883/T6069135) was synthesized as previously described^43^. [γ-^32^P]-ATP was purchased from ICN Biomedical (Aurora, OH). Antibodies to ROCKII, pSer19-LC20, pThr155-Prostate-apoptosis response (Par)-4, pSer82-Heat shock protein (HSP)-27, pSer3-cofilin, pThr508-LIMK1/pThr505-LIMK2 and LIMK1 were from Cell Signaling (Danvers, MA); LC20 and β-actin antibodies were from Santa Cruz Biotechnology (Dallas, TX); anti-pThr265-ZIPK was from Abcam (Cambridge, MA); anti-pThr696-MYPT1 and anti-pThr853-MYPT1 were from EMD Millipore/Upstate Biotechnology (Etobicoke, ON); anti-rabbit IgG coupled to horseradish peroxidase (HRP) was from Chemicon (Temecula, CA). The Enhanced Chemiluminescence (ECL) Kit was purchased from GE Healthcare (Piscataway, NJ), and the Phos-tag acrylamide reagent was from NARD Chemicals (Kobe City, Japan). All other chemicals were reagent grade and were obtained from Sigma Chemicals (Oakville, ON). Myosin regulatory light chains (LC20) were purified from chicken gizzard as previously described^45^. A constitutively-active form of ZIPK, GST-ZIPK(1–320), was expressed and purified with glutathione-Sepharose as previously described^25^. Constitutively-active recombinant human ROCKII(5–554) was obtained from SignalChem (Richmond, BC). Smooth muscle MLCK was purified from chicken gizzard as previously described^46^.

### Protein kinase assays

The phosphorylation of LC20 substrate peptide (KKKRPQRATSNVF) by ZIPK (10 μg/ml), ROCKII (4 μg/ml) or MLCK (4 μg/ml) was assayed at 25 °C in a final volume of 50 μl. The standard kinase reaction mixtures contained 25 mM HEPES buffer (pH, 7.4), 2 mM MgCl_2_, 200 μM ATP (containing 1 μCi [γ-^32^P]-ATP), and 100 μM LC20 peptide. MLCK reactions also contained purified bovine calmodulin (10 μg/mL) and 0.1 mM free CaCl_2_. Reactions were initiated by the addition of ATP solution and terminated after 15 min by spotting the mixture onto phosphocellulose P81 paper. After washing three times with 20 mM H_3_PO_4_, ^32^P incorporation was determined by scintillation counting. Kinetic parameters (K_m_, Vmax and K_*i*_) were determined using the Henri-Michaelis-Menten equation and Lineweaver-Burk plots. In some cases, the phosphorylation of LC20 protein by ZIPK or ROCKII was analysed by Phos-tag SDS-PAGE as previously described^14^.

### Cell culture and siRNA transfection

Human coronary artery smooth muscle cells (CASMC, CC-2583; Lonza; Allendale, NJ) were cultured as previously described^20^. Human ROCKII siRNA (sc-29474) and scrambled siRNA (sc-37007) were purchased from Santa Cruz Biotechnology. CASMCs were transfected with siRNAs using transfection reagent (sc-29528) according to the manufacturer’s protocol. Briefly, 2 × 10^5^ CASMCs were seeded in 2 ml antibiotic-free normal growth medium supplemented with FBS. Scrambled- and ROCKII-siRNAs were diluted with siRNA transfection medium (sc-36868) to give a final concentration of 20 nM. The mixture was gently overlaid onto the cells and incubated for 16 h at 37 °C. Normal growth medium (2 mL containing 2 times the normal serum and antibiotic concentrations) was added, and the cells were incubated for an additional 24 h. Fresh SmGM-2 growth medium was applied, and cells were harvested for analysis after 48 h.

### Immunocytochemistry

Cells were plated at 2 × 10^5^ in DMEM culture medium with 10% (v/v) FBS at 37 °C with 5% CO_2_. Cells were fixed in 4% (v/v) formaldehyde and then probed at 4 °C overnight with anti-pS3-cofilin (Thermo Scientific Pierce) diluted 1:100 in blocking serum. After washing with PBS, immunoreactivity was detected with goat anti-rabbit AlexaFluor488-conjugated secondary antibody. For F-actin visualization, AlexaFluor488-phalloidin (Molecular Probes) was diluted 1:40 in 1% (w/v) BSA in PBS and then incubated with the cells for 1 h at room temperature. Cells were rinsed with PBS, counterstained with DAPI for 5 min to detect nuclei, and then examined with an InCell 6000 Imaging System (GE Healthcare). For quantitation, the InCell 6000 Imaging System was programmed to complete whole-well scanning of four independent plates of cells. Ten random visual fields were analyzed from each well, and eight images were taken from each field. The F-actin fluorescence signal was quantified with ImageJ (https://imagej.nih.gov) by splitting the colour channels of the original image. The AlexaFluor488-phalloidin signal was identified by selecting cells of interest using the drawing tool and then applying the analysis parameters of the area and integrated density functions. For the pS3-cofilin signal, the intracellular immunofluorescence was quantified and then normalized to the DAPI nuclear counterstain.

### Western blot analyses

Whole cell extracts were prepared from CASMC cultures by first washing cells with PBS and then completing cellular lysis with addition of 2X sample buffer (2% (w/v) SDS, 100 mM DTT, 10% (v/v) glycerol, 0.01% (w/v) bromphenol blue, and 60 mM Tris-HCl, pH 6.8) with gentle rocking followed by heating at 95 °C for 5 min. Cellular extracts were resolved by SDS-PAGE and transferred to 0.2-μm nitrocellulose membranes in a Tris/glycine transfer buffer containing 10% (v/v) methanol. Non-specific binding sites were blocked with 5% (w/v) nonfat dry milk in Tris-buffered saline with Tween (TBST, 25 mM Tris-HCl, 137 mM NaCl, 3 mM KCl, and 0.05% (v/v) Tween-20). Membranes were washed and incubated overnight with primary antibody at 1:1,000 dilution with 1% (w/v) nonfat dry milk in TBST. Membranes were then incubated for 1 h with horseradish peroxidase (HRP)-conjugated secondary antibody (dilution 1:10,000) in TBST and developed with ECL reagent. LC20 phosphorylation was analyzed by Phos-tag SDS–PAGE as described previously^14^,^29^,^43^. After electrophoresis, proteins were transferred to polyvinylidene difluoride (PVDF) membranes at 25 V for 16 h at 4 °C. Proteins were fixed on the membrane with 0.5% (v/v) glutaraldehyde in phosphate-buffered saline (137 mM NaCl, 2.7 mM KCl, 10 mM Na_2_HPO_4_, and 1.76 mM KH_2_PO_4_) for 45 min and then washed with TBST. Nonspecific binding sites were blocked with 5% (w/v) nonfat dry milk in TBST. Membranes were washed with TBST and incubated overnight with anti-LC20 at a 1:1000 dilution in 1% (w/v) nonfat dry milk in TBST. Membranes were incubated for 1 h with HRP-conjugated secondary antibody (dilution 1:10,000) and developed with ECL reagent.

### Molecular docking

*In silico* docking experiments of DI with the ROCKII (PDB: 2F2U) and ZIPK (PDB: 3BQR) catalytic domain structures were completed with Arguslab software (v4.0.1; Planaria Software, Seattle, WA, http://www.arguslab.com). The Ascore algorithm was used to calculate the binding poses within a grid resolution of 0.4 Å. The docking search was completed without defining the target area or protein pocket, and the docking pose with the minimal energy was taken as the optimal binding mode.

### Data analyses

Data are presented as the mean ± S.E.M., with *n* indicating the number of independent experiments. Data were analyzed by Student’s t-test, and P < 0.05 was considered to indicate statistical significance. All statistical analyses were performed using the GraphPad Prism 6.0 program.

## Results

### The DI compound is a competitive inhibitor of ROCKII

**Figure 1:**
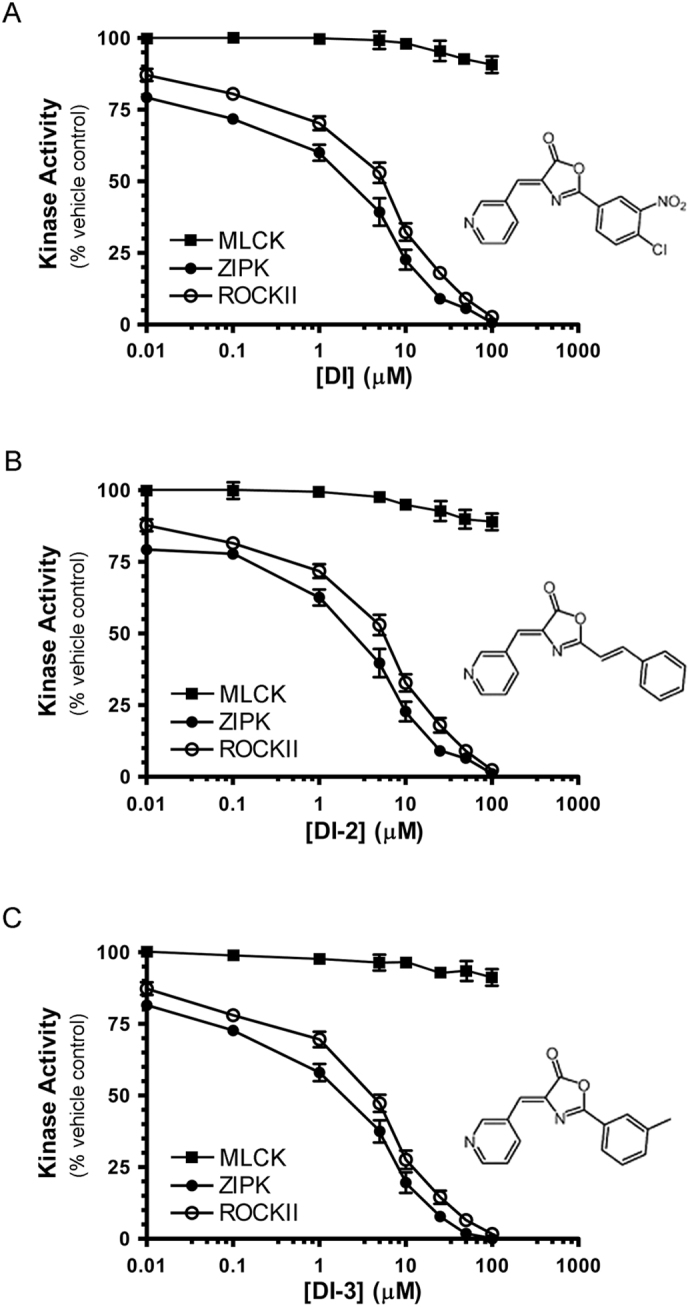
The effect of small molecule 5(4*H*)-oxazolone compounds on ZIPK, ROCKII, and MLCK activities *in vitro*. Purified recombinant ZIPK (0.5 μg), ROCKII (0.2 μg) and smooth muscle MLCK (0.18 μg) were incubated with [γ−^32^P] ATP solution and LC20 peptide substrate in the absence or presence of ZIPK/DAPK1 inhibitors: **(A)** DI, (4Z)-2-(4-chloro-3-nitrophenyl)-4-(pyridin-3-ylmethylidene)-1,3-oxazol-5-one; **(B)** DI-2, (4Z)-2-ethylidene(phenyl)-4-(pyridin-3-ylmethylidene)-1,3-oxazol-5-one; and **(C)** DI-3, (4Z)-2-(3-methylphenyl)-4-(pyridin-3-ylmethylidene)-1,3-oxazol-5-one. ^32^P incorporation into LC20 peptide was determined by scintillation counting and is reported as the % Activity relative to vehicle control. Data are means ± S.E.M. (*n* = 3). Where error bars are not present, they are smaller than the symbols.

**Figure 2:**
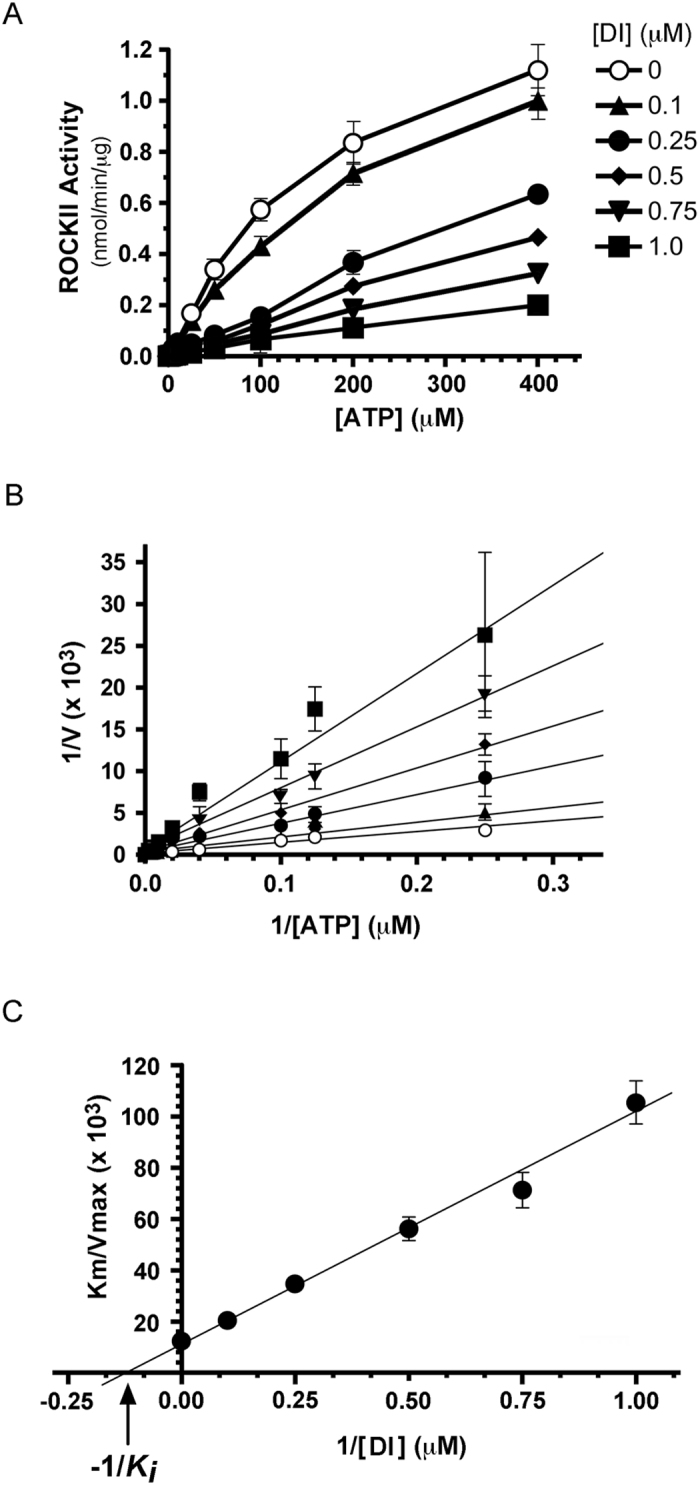
Kinetic analysis of (4Z)-2-(4-chloro-3-nitrophenyl)-4-(pyridin-3-ylmethylidene)-1,3-oxazol-5-one (DI compound) inhibition of ROCKII. (**A**) The effect of increasing DI concentration on the Michaelis-Menten kinetics of ROCKII for MgATP. The Km for ATP and Vmax values in the absence of DI compound were 31.3 μM and 1.3 nmol/min/μg, respectively. (**B**) Double-reciprocal plot of the rate of LC20 peptide phosphorylation (nmol/min/μg) as a function of ATP concentration reveals competitive inhibition of ROCKII activity with addition of increasing DI concentrations. (**C**) Secondary plot of apparent Km/Vmax for ROCKII(5-554) as a function of DI concentration provides a *K*_*i*_ value of 132 nM. Values indicate the mean ± S.E.M. (*n* = 5). Where error bars are not present, they are smaller than the symbols.

The (4Z)-2-(4-chloro-3-nitrophenyl)-4-(pyridin-3-ylmethylidene)-1,3-oxazol-5-one (DI) molecule was originally developed by a structure-based screening strategy as a potential therapeutic inhibitor for the treatment of ischemic reperfusion injury^41^. The DI compound was further characterized as a selective inhibitor of DAPK1 (IC50 = 69 nM) and DAPK3/ZIPK (IC50 = 225 nM)^42^ by screening a broad panel of kinases. However, during our preliminary application of DI to the study of ZIPK in smooth muscle calcium sensitization, we identified a potential off-target effect. We employed traditional ^32^P-phosphoryl transfer assays with LC20 peptide to examine the potential inhibitory effect of DI on contractile kinases, namely ZIPK, ROCKII and MLCK. A pronounced decrease in LC20 phosphorylation was detected upon treatment of constitutively-active ZIPK(1-320) and ROCKII(5-554) with increasing concentrations of DI (Fig. 1A). Similarly, two additional DI analogues with ZIPK and DAPK1 inhibitory potential that were previously disclosed by Okamoto and colleagues^41^,^42^, namely (4Z)-2-ethylidene (phenyl)-4-(pyridin-3-ylmethylidene)-1,3-oxazol-5-one (DI-2) and (4Z)-2-(3-methylphenyl)-4-(pyridin-3-ylmethylidene)-1,3-oxazol-5-one (DI-3), also inhibited the activity of ROCKII(5-554) (Fig. 1B,C, respectively). The application of increasing concentrations of DI compounds (0–100 μM) revealed no inhibition of MLCK. In addition, DI was found to have minor impact upon the activity of integrin-linked kinase (ILK) toward GSK-3 substrate, with inhibition observed only at high concentrations (>50 μM). ILK activity was about 60% of control levels at 100 μM (Supplementary Figure 1). A more detailed *in vitro* kinetic assessment of the DI compound was completed to confirm the inhibitory potency on ROCKII. In the absence of DI, the K_m_ for ATP and Vmax of ROCKII were determined to be 31.3 ± 1.1 μM and 1.3 ± 0.12 nmol/min/μg (Fig. 2A). The addition of different concentrations of DI (0–1 μM) increased the K_m_ without having any observable effect on the Vmax for ROCKII. A double-reciprocal plot confirmed that the DI compound acted as a competitive inhibitor of ROCKII with respect to ATP (Fig. 2B). In addition, a secondary plot of K_m_/Vmax as a function of [DI] was used to calculate a K_*i*_ value of 130 nM (Fig. 2C).

### Molecular docking studies identify putative binding sites and mode of binding for DI to ROCKII

**Figure 3:**
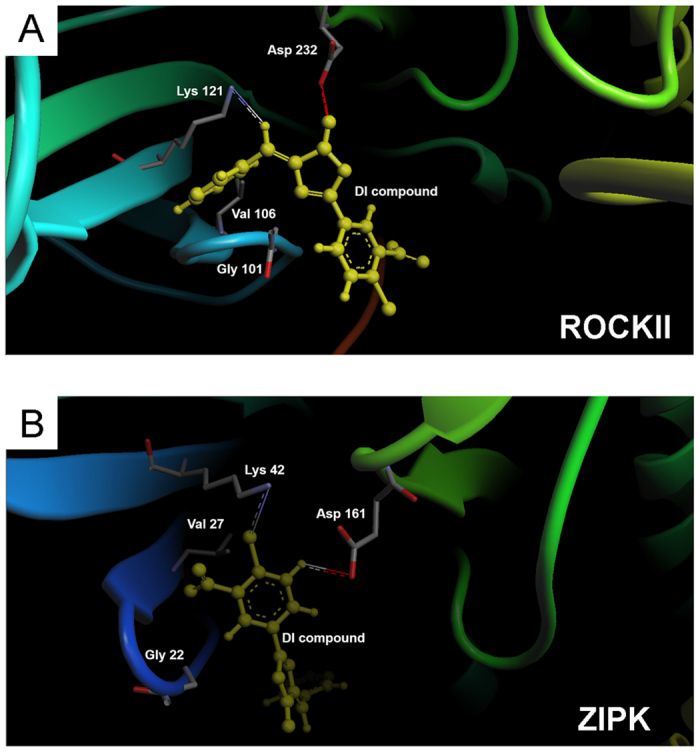
Computational model of DI inhibitor binding to ROCKII and ZIPK kinase domains. The lowest-energy binding mode was derived with Arguslab, and the predicted interaction surface of the DI compound and ATP-binding sites of ROCKII (**A**, PDB: 2F2U) and ZIPK (**B**, PDB: 3BQR) are shown. Key amino acid residues implicated in the interactions of ROCKII and ZIPK with DI are shown in the computational binding models.

As shown in Fig. 3A, the best pose for all possible binding configurations suggested thermodynamically stable binding of the DI ligand within the ATP-binding pocket of ROCKII (estimated free energy value of −8.18 kcal/mol). In addition, the computational docking studies revealed a similar minimal binding energy of −9.9 kcal/mol for DI binding to ZIPK (Fig. 3B). The computational binding models suggest that similar amino acid residues mediate interactions of ROCKII and ZIPK with the DI molecule: i) ROCKII, Lys121; ZIPK, Lys42: a conserved lysine residue that stabilizes ligand binding in the kinase domain by interacting with the α- and β-phosphate groups of ATP^47^, ii) ROCKII, Asp232; ZIPK, Asp161: the conserved residue of the DFG motif in the kinase domain that is required for Mg^2+^ coordination^47^, iii) ROCKII, Val106 and Gly99; ZIPK Val27 and Gly20: residues located in the glycine-rich loops (GXGXXGXV) of the kinase domain that interface with the phosphate groups of ATP^47^. Additional residues located within the glycine-rich loops (Gly101 and Ala102) and hydrophobic pocket (Phe103 and Phe106) of ROCKII were also implicated in DI binding.

### The DI compound inhibits ROCKII-induced LC20 phosphorylation *in vitro*

**Figure 4:**
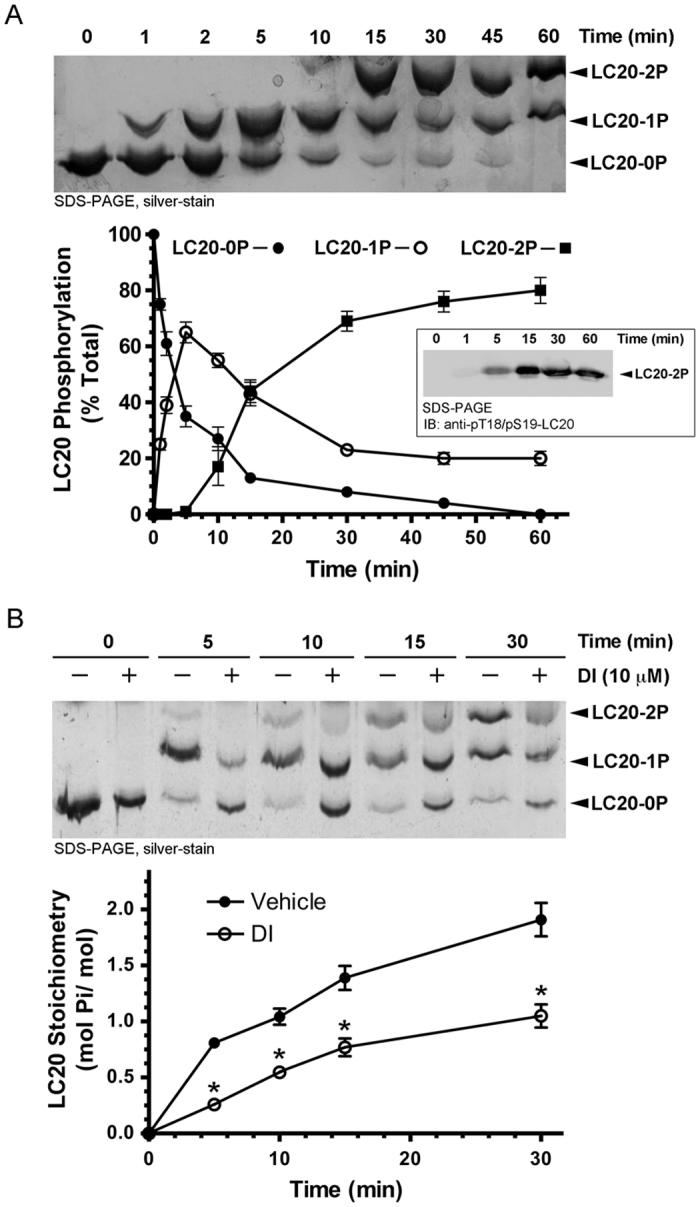
The DI compound attenuates the ROCKII-dependent phosphorylation of LC20 *in vitro.* (**A**) LC20 phosphorylation by ROCKII was analyzed by Phos-tag SDS-PAGE with detection of unphosphorylated (LC20-0P), mono (LC20-1P)-, and di (LC20-2P)-phosphorylated forms on a silver-stained SDS-PAGE gel. The various LC20 bands were quantified from the silver-stained gel by scanning densitometry and expressed as a percentage of the total LC20 signal. A representative silver-stained gel is shown above cumulative quantitative data. Inset: a representative western blot for LC20 phosphorylation by ROCKII confirmed diphosphorylation at T18 and S19. (**B**) A representative silver-stained SDS-PAGE gel and cumulative quantitative data are provided for the time course of LC20 phosphorylation by ROCKII in the presence of vehicle (DMSO) or DI (5 μM). LC20 phosphorylation stoichiometry is expressed as mol P_i_/mol LC20. *Significantly different from the corresponding value in the absence of DI (Student’s *t* test, p < 0.05). Values indicate the mean ± S.E.M. (*n* = 4).

LC20 mono- and di-phosphorylation by ROCKII were detected by Phos-tag SDS-PAGE with silver staining (Fig. 4A, upper panel). Phospho-specific antibodies were used to confirm the ROCKII-dependent diphosphorylation of LC20 at Ser19 and Thr18. Total LC20 phosphorylation was determined as % of the total LC20 (Fig. 4A, lower panel). DI (5 μM) inhibited LC20 diphosphorylation (Fig. 4B). Maximal LC20 phosphorylation stoichiometries (at 30 min) in the absence and presence of the DI compound were 1.91 ± 0.2 mol Pi/mol and 1.01 ± 0.07 mol Pi/mol, respectively (Fig. 4B, lower panel).

### Treatment of VSMCs with DI (or other ROCKII inhibitors) attenuates LC20, Par-4 and cofilin phosphorylation

**Figure 5:**
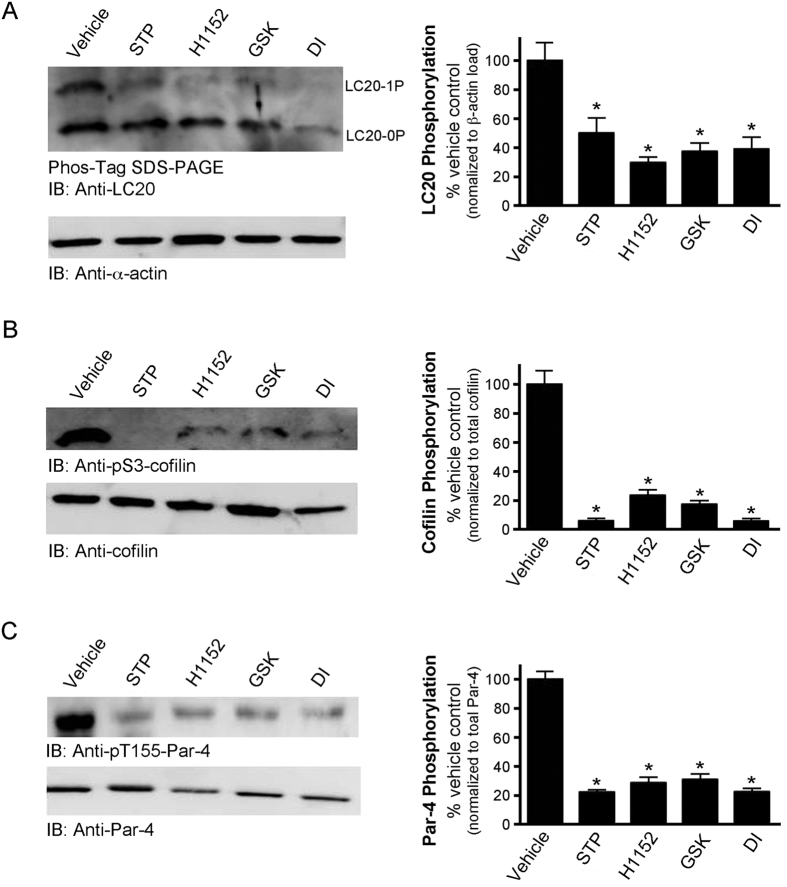
DI and known ROCKII inhibitors suppress the phosphorylation of LC20, cofilin and Par-4 proteins in vascular smooth muscle cells. Human coronary artery smooth muscle cells (passage 9) were serum-starved overnight and incubated with DI (50 μM), a broad-specificity kinase inhibitor (staurosporine (STP), 1 μM), specific ROCKII inhibitors (H1152, 50 μM; GSK269962A (GSK), 50 μM) or vehicle control (DMSO). (**A**) Phos-tag SDS-PAGE was used for the analysis of LC20 phosphorylation. The un (LC20-0P)- and mono (LC20-1P)- phosphorylated bands were detected by western blotting with anti-LC20, quantified by scanning densitometry and expressed as a percentage of the total LC20 signal. Phosphorylated cofilin (**B**) and Par-4 (**C**) were detected by western blotting with anti-pS3-cofilin and anti-pT155-Par-4, respectively. Bands were quantified by scanning densitometry and normalized to the loading control. Phosphorylation levels are expressed as a percentage of control (absence of inhibitors). Values represent means ± S.E.M. for *n* = 4 separate treatments. *Significantly different from vehicle control (Student’s *t* test, p < 0.05).

**Figure 6:**
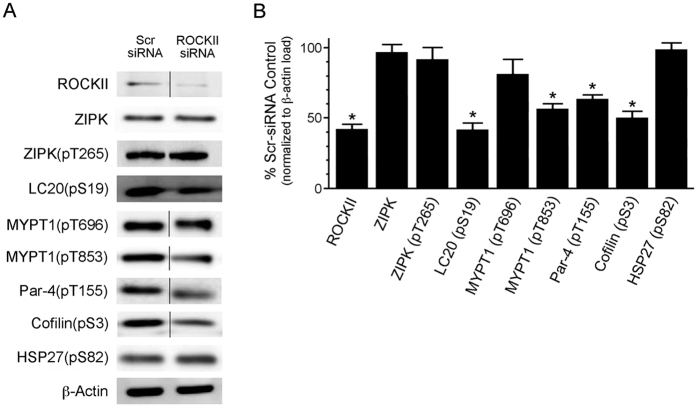
The effect of ROCKII knockdown by siRNA on the phosphorylation of various contractile regulatory proteins in vascular smooth muscle cells. Human coronary artery smooth muscle cells (passage 9) were treated with scrambled (Scr) siRNA or ROCKII siRNA for 48 h and then serum-starved overnight. (**A**) Whole cell lysates were collected and analyzed by western blotting for ROCKII and ZIPK expression as well as the phosphorylation of various proteins, including LC20 (pS19), ZIPK (pT265), Par-4 (pT155), MYPT1 (pT696 and pT853), cofilin (pS3), and HSP27 (pS82). (**B**) Bands were quantified by scanning densitometry and normalized to the β-actin loading control. Phosphorylation levels are expressed as a percentage of control (Scr-siRNA treatment). Values represent means ± S.E.M. for *n* = 4 separate experiments. *Significantly different from Scr-siRNA control (Student’s *t* test, p < 0.05).

Cultured CASMCs were used to examine the inhibitory effect of the DI compound on the phosphorylation of three ROCKII and/or ZIPK targets *in situ*. The Ser19 phosphorylation site of LC20 (targeted by both ZIPK and ROCKII^16^,^27^), the Thr155 phosphorylation site (a known ZIPK target^48^) of Par-4, and the inhibitory Ser3 phosphorylation site of the actin cytoskeleton depolymerizing protein cofilin (a known LIMK target downstream of ROCKII^21^) were examined. Other well-characterized ROCK inhibitors, H1152^49^ and GSK269962A^50^, were used for comparison purposes. Staurosporine was used as a positive control since this indolocarbazole molecule is associated with broadly-effective potency across the entire kinome^51^, including ZIPK and ROCK. The application of DI inhibitor significantly suppressed the phosphorylation of LC20 at Ser19 (Fig. 5A), cofilin at Ser3 (Fig. 5B) and Par-4 at Thr155 (Fig. 5C). Similar effects were observed when CASMCs were exposed to H1152 or GSK269962A. We also employed siRNA-mediated knockdown of ROCKII protein and evaluated its effect on substrate phosphorylation events in CASMCs. ROCKII-siRNA treatment suppressed ROCKII expression by ~60% when compared with scrambled siRNA (Fig. 6A), while no effect was observed on ZIPK expression or ZIPK Thr265 phosphorylation status. Interestingly, significant reductions in the phosphorylation status of LC20 (Ser19), Par-4 (Thr155), MYPT1 (at Thr853 but not Thr696), and cofilin (Ser3) were observed with ROCKII knockdown by siRNA (Fig. 6B). HSP27, which is known to be phosphorylated at Ser82 by protein kinase C^52^, was unaffected by ROCKII knockdown (Fig. 6B).

### The LIMK/cofilin pathway is targeted by the DI compound in VSMCs

**Figure 7:**
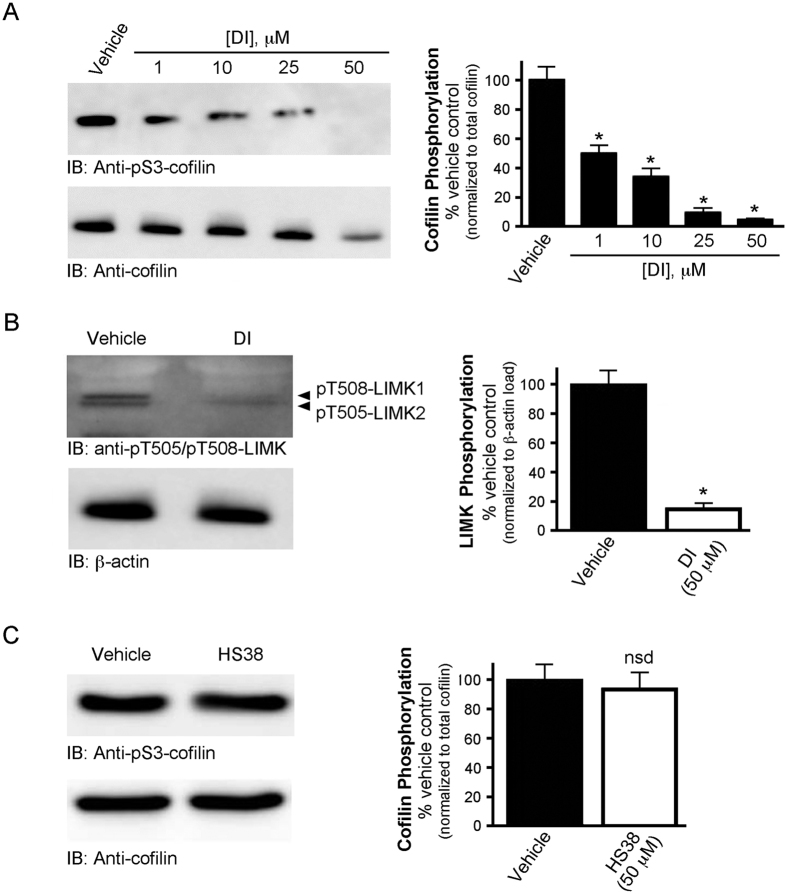
Cofilin and LIMK phosphorylations are attenuated in coronary artery smooth muscle cells with application of DI. **(A**) Human coronary artery smooth muscle cells (passage 10) were serum-starved overnight in the presence of increasing concentrations of DI (0–50 μM). Whole cell lysates were prepared, and phosphorylated cofilin was quantified by western blotting with anti-pS3-cofilin and normalized to total cofilin. (**B**) CASMCs were treated with vehicle or DI (50 μM); phosphorylated LIMK1/2 was quantified by western blotting with anti-pT505/pT508-LIMK and normalized to β-actin. (**C**) Phosphorylated cofilin was quantified by western blotting with anti-pS3-cofilin following treatment of CASMCs with the ZIPK inhibitor HS38 (50 μM). Values represent means ± S.E.M. for *n* = 4 separate treatments. Phosphorylation levels are expressed as a percentage of the vehicle control. *Significantly different from control; ^#^Significantly different from ROCKII-siRNA exposure (Student’s *t* test, p < 0.05); *nsd*- not significantly different.

**Figure 8:**
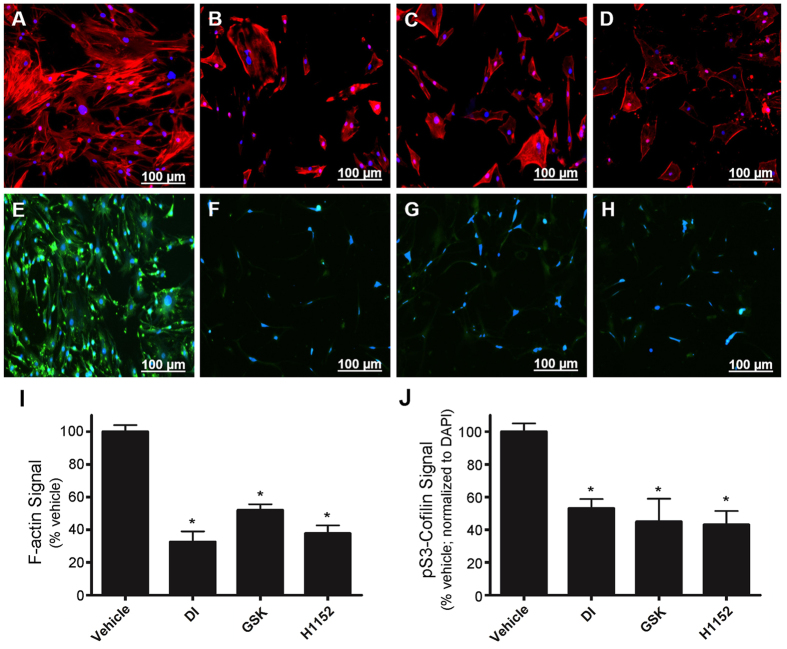
Immunocytochemistry of CASMCs for F-actin cytoskeleton architecture and pS3-cofilin levels following treatment with DI and known ROCKII inhibitors. Human coronary artery smooth muscle cells (passage 10) were serum-starved overnight in the presence of different ROCKII inhibitors. (**A–D**), CASMCs were fixed and stained with AlexaFluor488-phalloidin to examine reorganization of the F-actin cytoskeleton following vehicle control (DMSO; **A**), DI (50 μM; **B**), GSK269962A (50 μM; **C**), or H1152 (50 μM; **D**) treatments. (**E–H**), cofilin Ser3 phosphorylation was detected by immunocytochemistry following treatment of CASMCs with vehicle control (DMSO; **E**), DI (50 μM; **F**), GSK269962A (50 μM; **G**), or H1152 (50 μM; **H**). Representative images are shown with fluorescence signals for phalloidin-F-actin (red channel), pS3-cofilin immunoreactivity (green channel) and DAPI nuclear stain (blue channel). Scale bars = 100 μm. In (**I–J**), cellular fluorescence intensities were quantified. The total AlexaFluor488-phalloidin signal was identified by randomly selecting cells using the drawing tool and then applying the integrated density and area analysis parameters (**I**). For the quantification of pS3-cofilin fluorescence (**J**), the total cellular immunofluorescence was calculated and then normalized to the DAPI nuclear stain. Values represent means ± S.E.M. for *n* = 4 independent experiments. For each independent plate of cells, 10 random visual fields were acquired from each whole-well scan, and cells in 8 images were quantified from each field. *Significantly different from the vehicle control (Student’s t test, p < 0.05).

Cofilin activity is inhibited by phosphorylation of Ser3, an event that is mediated by LIMK acting downstream of ROCK^53^. A significant decrease in the phosphorylation of cofilin at Ser3 was observed with the application of increasing concentrations of DI (Fig. 7A). Moreover, treatment with DI was also found to attenuate the phosphorylation of pT508-LIMK1 and pT505-LIMK2 (Fig. 7B). Since additional kinases are known to activate LIMK and influence cofilin phosphorylation^53^, we also investigated if the attenuation of cofilin phosphorylation was triggered exclusively by the inhibition of ROCKII. In particular, the potential influence of ZIPK on the regulation of cofilin was interrogated because the protein kinase could also be inhibited by DI^41^,^42^. Another ATP-competitive ZIPK inhibitor (HS38; IC50 < 200 nM) does not target ROCKII *in vitro* when examined by ^33^P-ATP filter binding assay^43^ and does not inhibit LC20 phosphorylation by ROCKII when assessed with ^32^P-phosphoryl transfer assays^29^. The application of a similar concentration (50 μM) of the HS38 inhibitor revealed no significant changes in the phosphorylation levels of cofilin (Fig. 7C). These results suggest that the changes in cofilin phosphorylation upon administration of DI to CASMCs were exclusively associated with ROCKII and not ZIPK. Staining with phalloidin revealed remarkable changes in F-actin stress fiber architecture upon DI treatment of CASMCs when compared with the vehicle control (Fig. 8A,B,I). Cofilin Ser3 phosphorylation was also analyzed by immunocytochemistry in vehicle- and DI-treated cells (Fig. 8E,F,J). Immunostaining of anti-pS3-cofilin was significantly diminished in CASMCs after DI treatment. Similar effects on F-actin architecture and pS3-cofilin immunostaining were observed following treatment of CASMCs with known ROCK inhibitors: GSK269962A (Fig. 8C,G) and H1152 (Fig. 8D,H).

## Discussion

The (4Z)-2-(4-chloro-3-nitrophenyl)-4-(pyridin-3-ylmethylidene)-1,3-oxazol-5-one (DI) molecule is an ATP-competitive inhibitor developed by a structure-based screening strategy as a potential therapeutic agent to inhibit DAPK1 and ZIPK function(s). We set out to study the selectivity and potency of this compound to investigate the role of ZIPK in VSMC signaling pathways linked to cellular contractility and actin cytoskeletal dynamics. Surprisingly, the initial *in vitro* analyses revealed ROCKII as a prominent off-target for DI. Enzyme kinetic experiments determined the potency of the DI compound (Ki = 132 nM) toward ROCKII to be similar to that of Y27632 (Ki = 140 nM) and fasudil (Ki = 158 nM) and less than that of H1152 (Ki = 1.6 nM^49^) or GSK269962A (Ki = 4 nM^50^). Based on computational modeling, we conclude that the DI molecule has a similar binding mode in the ATP-binding pockets of ZIPK and ROCKII. This conclusion is supported by (i) the putative involvement of similar critical amino acids in DI binding to both kinases, and (ii) the predicted minimal binding energies for the interaction of DI with ZIPK and ROCKII. Together, the *in silico* modeling data and the empirical kinetic evidence indicate important off-target effects of DI against ROCKII. However, there is a discrepancy between our data and the original screening results presented by Okamato and colleagues^41^,^42^. The benzylidene oxazolone compounds selected by virtual screening were previously profiled for off-target effects using a Z’-LYTE Kinase Assay Kit with Ser/Thr 13 Peptide. A variety of kinases were tested, including ROCKII, and the compounds exhibited no significant inhibitory potential. The Z’-LYTE assay employs a FRET-based, coupled-enzyme protocol and is based on the differential sensitivity of phosphorylated and non-phosphorylated peptides to proteolytic cleavage^54^. The assay involves three main steps: (1) a phosphorylation reaction is triggered by normal kinase activity and phosphoryl transfer from ATP to the peptide substrate; (2) site specific proteolysis of nonphosphorylated peptides liberates FRET activity; and (3) peptide phosphorylation is determined by calculating the relative FRET activity since phosphorylated substrate peptide is resistant to cleavage and disruption of the FRET activity, whereas uncleaved phosphorylated peptides maintain FRET. However, there are multiple limitations associated with this platform, including (i) low efficiency as a single peptide substrate was used to screen DI against a large panel of different kinases with variable consensus recognition motifs; and (ii) the indirect nature of phosphorylation assessment based on the proteolytic efficacy for mono-, di-phosphorylated and non-phosphorylated substrate pools. One or both of these issues likely contributed to the false negative result for DI against ROCK. Our kinetic assessment of DI and ROCKII provided herein was completed with the gold standard assay of ^32^P-phosphoryl transfer and direct quantification of radiolabeled substrate with scintillation counting. The results confirm the inhibitory potency of the benzylidene oxazolone compounds toward both ZIPK and ROCKII.

The first reported small molecule inhibitor of the DAPK family was based on an aminopyridazine scaffold^55^. Although its potency was in the μM range, a single injection of this inhibitor attenuated brain tissue damage when administered before or 6 h after ischemic injury. Subsequently, peptide inhibitors derived from the autoinhibitory domain of ZIPK were also characterized^56^. The application of this peptide inhibitor to VSM was not satisfactory due to a lack of consistency in data obtained from different smooth muscle types^57^. Following this, a second set of small molecule inhibitors of DAPK1/ZIPK, the benzylidene oxazolone compounds that include DI, was reported^41^,^42^. Although they displayed potencies with IC50s ranging from 250 to 580 nM, the electrophilic and amine reactive nature of benzylidine oxazolones raises concerns about their stability in a biological milieu. A third class of small molecule inhibitors against ZIPK also display nM potencies^40^; however, these oxo-β-carboline derivatives also possess broad inhibitory profiles against a large number of other non-related protein kinases. A fourth scaffold, thiol-substituted pyrazolo[3,4-*d*]pyrimidines (e.g., HS38) display similar or greater potencies versus DAPK1/ZIPK and do not display any of the off-target liabilities of other published DAPK inhibitors against contractile kinases such as ROCKII^43^. The structural components of HS38 at the thioether and aryl regions around the pyrazolo[3,4-*d*]pyrimidinone core are important for DAPK/ZIPK affinities and are thought to be sufficient to distinguish the selectivity profile of HS38 from other DAPK/ZIPK inhibitor scaffolds. Although HS38 is not associated with any compromising effects on ROCKII activity, the molecule does possess non-selective effects toward the PIM kinases and IRAK4 that must be considered^43^. As the pharmacological landscape for small molecule inhibitors of ZIPK continues to mature, it is important to stress cautionary statements previously presented in the literature regarding the specificity and utility of protein kinase inhibitors^58^,^59^. In this regard, the specificities of protein kinase inhibitors cannot be assessed simply by studying their effect on kinase members that are closely related in primary structure, and it will be critical to ensure that similar biological responses are observed following treatment with at least two structurally unrelated inhibitors of ZIPK.

The data presented herein reveal DI to possess similar efficacy toward both ZIPK and ROCKII activities; nevertheless, the DI compound has been used in two recent studies by Usui and colleagues to assess the role of ZIPK in cardiovascular disease^31^,^44^. In the first study, the *in vivo* administration of DI to spontaneously-hypertensive rats was associated with decreases in blood pressure, reactive oxygen species (ROS) production, inflammatory responses, as well as vascular smooth muscle contractility and hypertrophy^31^. *In vitro* treatment of human umbilical vein endothelial cells with DI could also suppress tumor necrosis factor (TNF)α-induced inflammatory responses via ROS-dependent mechanisms. In a second study, the DI compound was used *in vitro* to link ZIPK with PDGF-BB-induced vascular SMC proliferation and migration through p38MAPK and HSP27 pathways^44^. Additional *in vivo* treatments with DI were shown to attenuate vascular structural remodeling during carotid neointimal hyperplasia. Both studies provide descriptions of unique roles for ZIPK in smooth muscle and signaling cascades leading to modulation of downstream targets, and in some cases, the influence of ZIPK was also validated *in vitro* with siRNA knock-down experiments. For example, TNFα-dependent effects on vascular cell adhesion molecule (VCAM)-1 expression, ROS production, and p38MAPK, Akt and JNK signaling were also linked to ZIPK with small interfering RNA treatments.

Regrettably, the concurrent inhibition of ROCKII during DI treatment could provide an equally rational mechanism for some *in vivo* outcomes. These two studies of DI and ZIPK inhibition in vascular smooth muscle systems profiled the phosphorylation status of key regulatory proteins. TNFα activation was linked to ZIPK-dependent effects on VCAM-1, E-selectin, cyclooxygenase (COX)-2, as well as ROS production, LC20 phosphorylation, and cellular migration of endothelial cells. Some of these proteins have been previously identified as downstream targets of RhoA/ROCKII signaling. For example, previous reports suggest that the activation of ROCK during TNFα-induced inflammation in lung endothelial cells leads to increased LC20 phosphorylation and cellular permeability^60^. Moreover, another study showed a significant attenuation of endothelial cell adhesion after the application of the ROCKII inhibitor, fasudil (HA1077), with reductions in the endothelial expression of VCAM-1 and decline in vascular inflammation^61^. The relationship between ROCKII and COX-2 was previously investigated with results linking ROCKII with the stimulation of COX-2, and subsequent reduction of E-cadherin and α-catenin along with disruption of adherens junction formation and increased cellular motility^62^. Experimental approaches that applied both siRNA and DI treatments provided links between ZIPK and a group of downstream targets (HSP27, ERK1/2 and p38MAPK) involved in reorganization of the actin cytoskeleton during vascular smooth muscle remodeling^44^. But, there have been previously described linkages between PDGF-BB-induced SMC hypertrophy and ROCK signaling in studies employing Y27632^63^. In addition, the involvement of ROCK/LIMK/cofilin pathways in cytoskeletal dynamics has been comprehensively described^15^,^22^. It was previously proposed that HSP27 was acting downstream of ZIPK to influence cytoskeletal dynamics. We did not detect any changes in the key phosphorylation site Ser82 of HSP27 using a ROCKII siRNA approach, so further analysis of the linkage between ZIPK and HSP27 phosphorylation in VSMCs will require more investigation. Taken together, there are numerous difficulties in the description of ZIPK substrates in smooth muscle cells/tissue due to the utilization of an inhibitor (DI) with at least one relevant off-target effector (ROCKII). As such, it is difficult to identify whether the changes in vascular inflammation markers observed with *in vivo* DI treatment are restricted to ZIPK, a secondary effect of ROCKII signal attenuation, or some combination of the two signaling pathways.

A complex integration of cytoskeletal events coincident with the activation of the actomyosin system regulates contractile force development in VSMCs. We have employed small interference RNA to knockdown ROCKII in CASMCs and identified diminution in the phosphorylation status of LC20 at Ser19, MYPT1 at the inhibitory Thr853 site (but not Thr696), and cofilin at Ser3. These data were consistent with previously defined roles for ROCKII in VSMCs^15^. We also demonstrated that treatment of VSMCs with DI (and other ROCKII inhibitors) could retard the phosphorylation of LC20, Par-4 and cofilin. Utilization of HS38 in VSMCs showed no effect on cofilin phosphorylation, suggesting that its phosphorylation was exclusively inhibited by ROCKII and not ZIPK. Furthermore, our data also revealed the potential for novel regulation of Par-4 phosphorylation by ROCKII. Current evidence supports a role for ZIPK-induced phosphorylation of Par-4 at Thr155^28^; however, Par-4 phosphorylation was not affected by application of the ZIPK inhibitor HS38 to VSM^29^. The attenuation of Par-4 Thr155 phosphorylation upon ROCKII siRNA treatment raises the question if ROCKII regulates Par-4 phosphorylation status. Further investigation will be required to assess if any links between ROCKII and Par-4 exist in VSMCs.

The new availability of small molecule inhibitors provides an opportunity to examine aspects of ZIPK signaling in VSM that were previously not attainable. While some kinase inhibitors are highly specific and will only recognize a restricted number of target molecules *in vivo*, others have broad target specificity and can impact upon multiple targets and regulate diverse cellular processes^58^,^59^. Unfortunately, the interpretation of results for the pharmacological inhibition of ZIPK with DI is compromised by overlapping inhibitory efficacy against another protein kinase that possesses similar functional contributions to smooth muscle contraction, namely ROCKII. Significantly, our studies indicate that the DI compound will not allow for distinctions between ZIPK and ROCKII signaling in VSMCs.

## Additional Information

**How to cite this article**: Al-Ghabkari, A. *et al*. A novel inhibitory effect of oxazol-5-one compounds on ROCKII signaling in human coronary artery vascular smooth muscle cells. *Sci. Rep.* **6**, 32118; doi: 10.1038/srep32118 (2016).

## Supplementary Material

Supplementary Information
