# Supplementary material for: A novel inhibitory effect of oxazol-5-one compounds on ROCKII signaling in human coronary artery vascular smooth muscle cells
Source: Sci Rep. 2016 Aug 30;6:32118. doi: 10.1038/srep32118 (PMC5004178; doi:10.1038/srep32118)
Supplement: Supplementary Information [file srep32118-s1.doc]

**Supplemental Materials for:**

A novel inhibitory effect of oxazol-5-one compounds on ROCKII signaling in human coronary artery vascular smooth muscle cells

Abdulhameed Al-Ghabkari^1^, Jing-Ti Deng^1^, Paul C. McDonald^2^, Shoukat Dedhar^2^, Mana Alshehri^1^, Michael P. Walsh^1^ and Justin A. MacDonald^1^

^1^Department of Biochemistry & Molecular Biology, University of Calgary,

3280 Hospital Drive NW, Calgary, AB, T2N 4Z6, Canada

^2^Department of Integrative Oncology, BC Cancer Research Centre, 675 West 10^th^ Ave, Vancouver, BC, V5Z 1L3, Canada


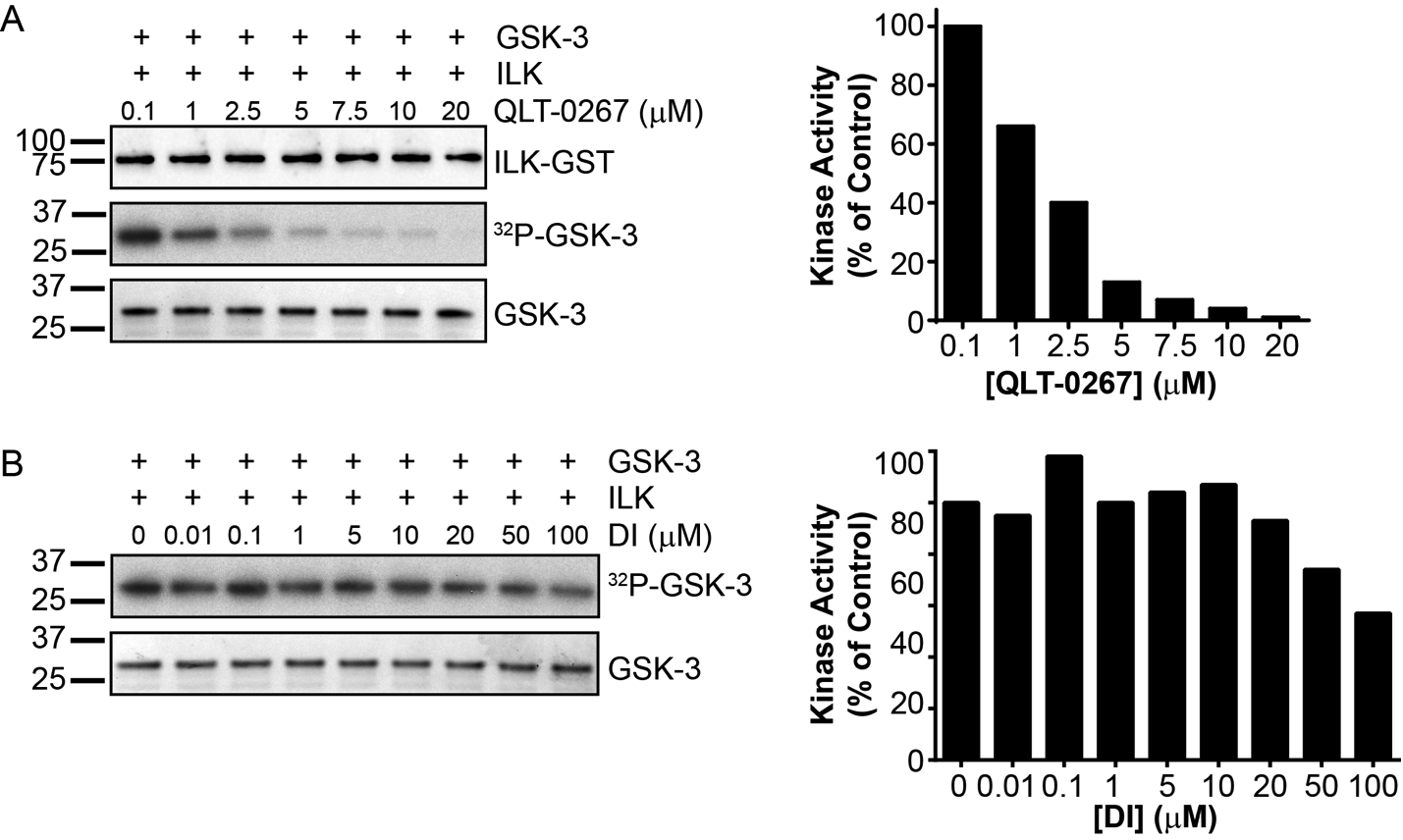


**Supplementary Figure 1. The effect of the small molecule DI on ILK activity *in vitro*.** Purified recombinant ILK (0.67 μM) was incubated with [γ-^32^P] ATP solution and GSK-3 peptide substrate in the presence or absence of the specific inhibitor of ILK, QLT-0267 **(A)** and the ZIPK/DAPK1 inhibitor, DI **(B)**. ^32^P incorporation into GSK-3 substrate was determined by autoradiography, total GSK-3 was detected by Coomassie staining and ILK was assessed by Western blot (left panel). ^32^P incorporation into GSK-3 was normalized to total amounts of GSK-3 and is reported as the % activity relative to control (right panel).

**Supplementary Materials and Methods**

**Integrin-linked kinase (ILK) activity assay.** The ILK kinase assay was performed as described previously (Maydan et al, 2010 PLoS One 23;5(8):e12356). Reactions containing purifed, recombinant GST-tagged ILK (0.67 μM, 50 ng/20 μl reaction; SignalChem Lifesciences Inc, Richmond, BC, Canada) and the ILK substrate, glycogen synthase kinase-3 (GSK-3; 2.8 μM, 1.5 μg/20 μl reaction; Cell Signaling Technology Inc, Danvers, MA, USA), were carried out using standard conditions. Stock solutions of the specific inhibitor of ILK, QLT-0267 (25 mM in DMSO) and the ZIPK/DAPK1 inhibitor, DI (5 mM in DMSO) were diluted to appropriate concentrations in water and were added immediately prior to initiation of the reaction. Kinase reactions were initiated by the addition of 10 μCi [γ-^32^P] ATP (Perkin-Elmer, Woodbridge, ON, Canada) and terminated with the addition of sample buffer. Proteins were then separated by SDS-PAGE and transferred to nitrocellulose. Alternatively, gels were stained by Coomassie blue. ^32^P incorporation into GSK-3 substrate was determined by autoradiography and ILK was assessed by western blot. Total GSK-3 was assessed by Coomassie blue staining. Signal intensities were quantified by densitometry using ImageJ 1.45s software. ^32^P incorporation into GSK-3 was normalized to total amounts of GSK-3 and is reported as the % activity relative to control.

**References**

Maydan, M., McDonald, P.C., Sanghera, J., Yan, J., Rallis, C., Pinchin, S., Hannigan, G.E., Foster, L.J., Ish-Horowicz, D., Walsh, M.P. & Dedhar, S., Integrin-linked kinase is a functional Mn2+-dependent protein kinase that regulates glycogen synthase kinase-3beta (GSK-3beta) phosphorylation. *PLoS One* 5 8, e12356 (2010).
